# Polysaccharides from Discarded Stems of *Trollius chinensis* Bunge Elicit Promising Potential in Cosmetic Industry: Characterization, Moisture Retention and Antioxidant Activity

**DOI:** 10.3390/molecules28073114

**Published:** 2023-03-30

**Authors:** Yang Liu, Qiwei Guo, Saimin Zhang, Yilin Bao, Mengling Chen, Lin Gao, Yang Zhang, Hongli Zhou

**Affiliations:** 1 School of Biology and Food Engineering, Changshu Institute of Technology, No. 99, Nan Sanhuan Road, Changshu 215500, China; 2 College of Chemical and Pharmaceutical Engineering, Jilin Institute of Chemical Technology, No. 45 Chengde Road, Longtan District, Jilin 132022, China

**Keywords:** *Trollius chinensis* Bunge, polysaccharide, moisture retention, antioxidation

## Abstract

Unconventional polysaccharides as representative active substances from stems of *Trollius chinensis* Bunge (TC) were studied. Crude polysaccharides from the stems of TC (TCSP) and the petals of TC (TCPP) were extracted, and the moisture retention and antioxidation activities of both TCSP and TCPP in vitro were studied. The weight-average molar masses (M_w_) of TCSP (6.07 × 10^5^ Da) were lower than those of TCPP (9.72 × 10^5^ Da). Glucuronic acid and xylose only existed in TCSP, and the molar ratio of galacturonic acid and mannose in TCSP was significantly higher than that in TCPP. No significant differences in moisture retention ability were found between TCSP and TCPP. The reducing capacity and dphenyl picryl hydrazinyl (DPPH) radical scavenging capacity of TCSP were slightly weaker than those of TCPP. The 2,2-azino-bis (3-ethylbenzothiazoline–6-sulfonic acid) (ABTS) radical scavenging capacity of TCSP can be equivalent to that of TCPP. The moisture retention ability was not different between TCSP and TCPP, which are both highly homologous with traditional humectants. The antioxidation assays in vitro demonstrated that the antioxidant activity of TCSP is stronger compared to that of some plant-derived polysaccharides. The stems of TC can be a promising source of unconventional polysaccharides, which possess moisture retention and antioxidation capacities for the cosmetics industry.

## 1. Introduction

*Trollius chinensis* Bunge (TC), belonging to *Ranunculaceae* and with a plant height of 30–100 cm, often grows on grass slopes or mountains (at altitude of 1000–2200 m) [1]. Medicinal records of TC were firstly found in *A Supplement to the Compendium of Materia Medica*, an ancient Chinese medical book that was compiled by Zhao Xuemin in 1765 [2]. Modern pharmacological research has confirmed the pharmacological benefits of TC in treating colds, respiratory infections, tonsillitis, pharyngitis and so on [3,4,5]. Some researchers attribute the pharmacological effects of TC to its various active substances, such as flavonoids, polysaccharides, and organic acids [6,7,8]. In 2003, TC was selected as a compound antiatypical-pneumonia-compatible-drug and played an important role in the outbreak of severe acute respiratory syndrome (SARS) in China [9]. Similar active substances are present in the petals and stems of TC. Moisturizing, as a basic function of cosmetics to prevent water evaporation in skin and improve cell activity, is also an important part of skin care [10,11]. Traditionally, humectants are not used alone, but are often combined with other humectants, especially natural humectants [12,13]. For example, hyaluronic acid (HA), an animal polysaccharide, was first extracted from bovine vitreous in 1934 [14], and was recognized as the best moisturizing factor in the cosmetics industry. However, wide application of HA in the cosmetics industry is difficult and limited by the low access to it and its high cost. Accordingly, plant polysaccharides are rich in hydrophilic hydroxyl and carboxyl groups and exhibit some excellent physical and chemical properties, such as strong water absorption, high viscosity and high film-forming ability [15,16]. Hence, plant polysaccharides are developed continuously in cosmetics and have exhibited great development potential in recent years. 

Polysaccharides have been tested as moisturizing ingredients in cosmetic products in some kinds of plants, such as *Barbados aloe* [17], *Bletilla striata* [18], and *Dendrobium huoshanense* [19]. Additionally, researchers treat plant polysaccharides as highly promising sources of antioxidants because polysaccharides have strong ability to scavenge free radicals [20,21]. However, the plant polysaccharides used as humectants in cosmetics mostly come from herbal medicine, leading to an increase in product costs and prices. Indeed, the stems of TC (TCS) may be one excellent plant source of polysaccharides. It is confirmed that some types and contents of active substances (e.g., flavonoids and polysaccharides) are close to or even higher than those in petals of TC (TCPP) [22].

This study aimed to investigate promising unconventional polysaccharides as representative active substances from TCS and discuss their potential application in the cosmetics industry to decrease costs and avoid waste. Furthermore, polysaccharides extracted by a water-extraction method were analyzed via monosaccharides composition and infrared analyses. Moreover, the moisture retention and antioxidation activities in vitro of polysaccharides from TCS (TCSP) were studied. As a contrast, the structure, moisture retention and antioxidation activities of polysaccharides from TC petals (TCPP) were also evaluated. This study may provide a promising unconventional polysaccharide from TCS as a humectant for the cosmetic industry, and also provide effective ways to improve the comprehensive utilization of TC.

## 2. Results

### 2.1. Yields and Main Chemical Compositions of Polysaccharides from TCS and TCP 

The contents of total sugars, uronic acid, flavonoids, and proteins in TCSP and TCPP are shown in Table 1.

No difference was found between the yields of TCSP and TCPP. The total sugar contents of TCSP and TCPP are 56.99 ± 8.12% and 46.09 ± 1.68%, respectively. It is well-known that flavonoids are more soluble in organic solvents. Most flavonoids should be removed during the processes of water extraction, alcohol precipitation, and dialysis. However, it is worth noting that the flavonoid content in TCSP and TCPP are 8.06 ± 1.82% and 9.52 ± 3.42%, respectively. Therefore, we speculate that the flavonoids in the sample may have linked with polysaccharides to form glycosides, and this may be the typical characteristic of the polysaccharide structure of Trollius chinensis. Some studies confirm the existence of multiple flavonoids in polysaccharides and provide necessary clues for the above speculation [5,10]. The contents of uronic acid in TCSP and TCPP indicate that TCSP and TCPP may belong to acid polysaccharides [23]. The protein contents are both lower than 1%, proving that protein removal is more thorough. 

### 2.2. Molecular Weights and Monosaccharide Composition of TCSP and TCPP

The number-average molecular weight (M_n_) of TCSP is significantly smaller (1.71 × 10^5^ Da) than that of TCPP (7.33 × 10^5^ Da) (Table 2). The weight-average molar masses (M_w_) of TCSP and TCPP are 6.07 × 10^5^ and 9.72 × 10^5^ Da, respectively. Although the M_w_ of TCSP is close to that of TCPP, the median molecular weight of TCSP is still lower. The poly-dispersion index (PDI, 3.55) of TCSP shows a wide molecular weight distribution, which is different from the PDI of TCPP. 

The monosaccharide compositions of TCSP and TCPP are displayed in Figure S1 and Table 2. Clearly, TCSP and TCPP showed similar monosaccharide profiles, including mannose (Man), rhamnose (Rha), galacturonic acid (GalA), glucose (Glu), galactose (Gal), arabinose (Ara) and fucose (Fuc) (Figure S1). Interestingly, glucuronic acid (GlcA) and xylose (Xyl) only exist in TCSP, but not in TCPP. Obviously, the molar ratios of GlcA and Man in TCSP are significantly higher than those in TCPP, and the molar ratios of other monosaccharides in TCSP are lower than those in TCPP. Both TCSP and TCPP may be acidic polysaccharides owing to the relative high content of GalA. Fuc may be a notable monosaccharide in both TCSP and TCPP. This result is slightly different from the polysaccharide and monosaccharide composition of *Trollius chinensis* Bunge in Hebei Province, China [24], which may be ascribed to the different growing regions of the plants. Fuc is a rare sugar mainly found in natural plants, such as seaweeds and gums. In addition to a certain moisturizing effect, Fuc also has an antiaging effect via increasing skin thickness, accelerating skin tissue regeneration, reducing wrinkles and raising skin elasticity [25,26]. Because the contents of Man, Rha, Glu, Gal, Ara and Fuc in TCPP are higher than those in TCSP, the activities of TCPP and TCSP may be different.

### 2.3. Fourier Transform Infrared Spectrometry (FT-IR) Analysis of TCSP and TCPP 

**Figure 1:**
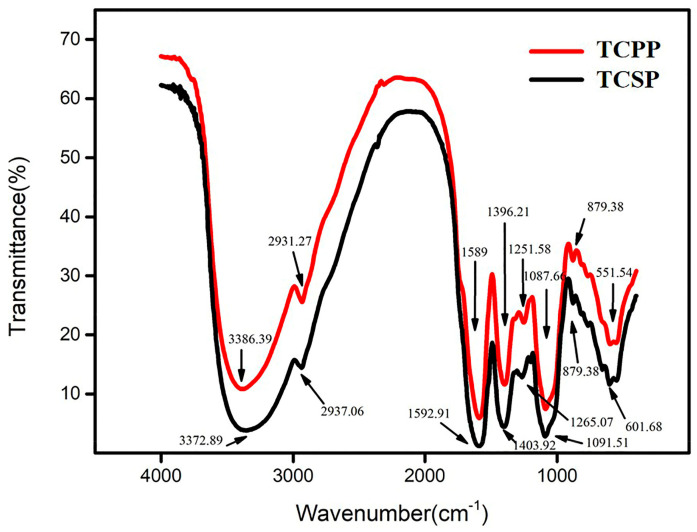
Fourier transform infrared spectrometry spectrum of TCSP and TCPP. TCSP: polysaccharides from stems of *Trollius chinensis* Bunge; TCPP: polysaccharides from petals of *Trollius chinensis* Bunge.

Figure 1 displays the characteristic absorption peaks of TCPP and TCSP. 

For the TCSP, the wide, pure and intense peaks within 3600–3200 cm^−1^ correspond to the stretching vibration of hydroxyl groups (O-H) [27]. It is speculated that this is a polysaccharide structure of multi-molecular association with inter-molecular hydrogen bonding, and there may be a chelate bond of C=O in the molecules [28]. Moreover, the absorption peak at 2933 cm^−1^ is ascribed to the C-H stretching vibration [29]. The weak peak at 2312 cm^−1^ is the stretching vibration of C=C, which may prompt the presence of an unsaturated bond in the polysaccharide structure. The strong peak at 1589 cm^−1^ indicating the bending of C=O and the band around 1396 reflecting the banding of C-H are both ascribed to the carboxylic acid group [30]. The bands around 1252 and 1088 cm^−1^ are assigned to the asymmetrical S=O stretching vibration and C-O stretching vibration, respectively. TCSP may belong to sulfated polysaccharides, which may prompt the potential capacity for antioxidation [16]. The characteristic absorption peaks of TCPP are similar to those of TCSP. Nevertheless, unlike TCSP, there is an absorbance peak at 657 cm^−1^, which may indicate the bending-out of O-H. These spectral findings prove that the structure and active abilities of TCSP may be homologous with those of TCPP.

### 2.4. Moisture Absorption and Retention of TCSP and TCPP

**Figure 2:**
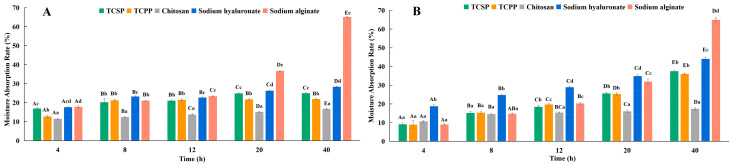
Moisture absorption of TCSP, TCPP, chitosan, sodium hyaluronate and sodium alginate for indicated times, A (81% RH) and B (43% RH). Capital letters represent inter-group differences, and lowercase letters represent intra-group differences. TCSP: polysaccharides from stems of *Trollius chinensis* Bunge; TCPP: polysaccharides from petals of *Trollius chinensis* Bunge.

The moisture absorption rate (Ra) of all the samples continuously increased in the indicated time at 81% relative humidity (RH) (Figure 2A) and at 43% RH (Figure 2B). The Ra of sodium alginate rose much faster than that of other samples. However, other samples showed an obvious uptrend until 20 h, and then showed a slight uptrend. The Ra of TCSP (37.45 ± 0.59%) and TCPP (36.05 ± 0.55%) increased in a similar trend (*p* > 0.05), and rose faster than chitosan (17.34 ± 0.52%, *p* < 0.05), and slower than sodium hyaluronate (44.07 ± 0.83%, *p* < 0.05) at 81% RH after exposure for 40 h. The Ra of sodium alginate and chitosan also continuously increased for the indicated time at 43% RH, which was similar to the trends at 81% RH. However, after exposure for 40 h, the Ra values of TCPP (21.86 ± 0.31%), TCSP (25.01 ± 0.16%) and hyaluronate (28.36 ± 0.26%) were lower (*p* < 0.05) than those at 81% RH, and did not change significantly after 20 h. 

**Figure 3:**
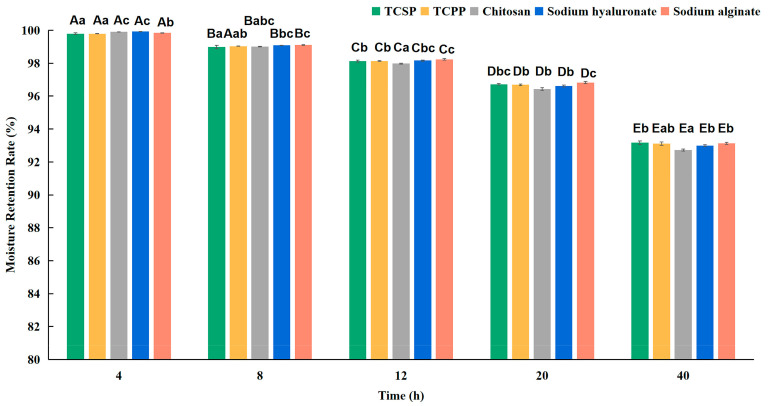
Moisture retention of TCSP, TCPP, chitosan, sodium hyaluronate and sodium alginate for indicated times (43% RH). Capital letters represent inter-group differences, and lowercase letters represent intra-group differences. TCSP: polysaccharides from stems of *Trollius chinensis* Bunge; TCPP: polysaccharides from petals of *Trollius chinensis* Bunge.

The moisture retention (Rh) of all samples shown in Figure 3 demonstrates an obvious downtrend in the indicated time. There is no significant difference (*p* > 0.05) in Rh among the samples. After exposure for 40 h at 43% RH, the Rh values of TCSP and TCPP are slightly higher compared with those of chisan, sodium hyaluronate and sodium alginate. 

There is no difference between the Ra values of TCPP and TCSP (*p* > 0.05), which are both higher than those of chitosan, and lower than those of sodium alginate and sodium hyaluronate at 81% RH. In comparison, the Ra of TCSP is slightly higher than that of TCPP (*p* < 0.05), but that of both are higher than that of chitosan, lower than that of sodium alginate, and close to that of sodium hyaluronate at 43% RH. Compared with chitosan, sodium hyaluronate and sodium alginate, both TCSP and TCPP manifest stronger moisture retention ability at 43% RH. Meanwhile, TCSP and TCPP showed strong moisture absorption and retention, compared with some plant polysaccharides, including *Dendrobium huoshanense* [19], and *Rosa rugosa* petals [31].

These results prove the huge potential of TCSP as a promising humectant for the cosmetics industry. From the molecular perspective, sodium alginate has perfect moisture absorption ability mainly because the alginate polysaccharides are composed of guluronic and mannuronic acid units and possess gel-forming ability through cation binding [32]. Hyaluronic acid is a biomacromolecule composed of glucuronic acid and acetylglucosamine, with a molecular weight of about 1−40 × 10^5^ Da. The structure endows it with a good-enough film-forming effect, so it can easily form a grid structure to enable the moisturizing effect [14]. Based on the analysis of the main compositions, monosaccharides and Fourier transform infrared spectrometry (FT-IR), the TCPP and TCSP demonstrate relative high moisture absorption ability probably because of the hydroxyl and carboxyl in GalA and Fuc. These polar groups in TCSP may form a hydrogen bond with H_2_O to intercept water molecules in the skin for moisture retention [17,33]. 

### 2.5. Antioxidant Activities of TCSP and TCPP 

#### 2.5.1. Reducing Capacity

**Figure 4:**
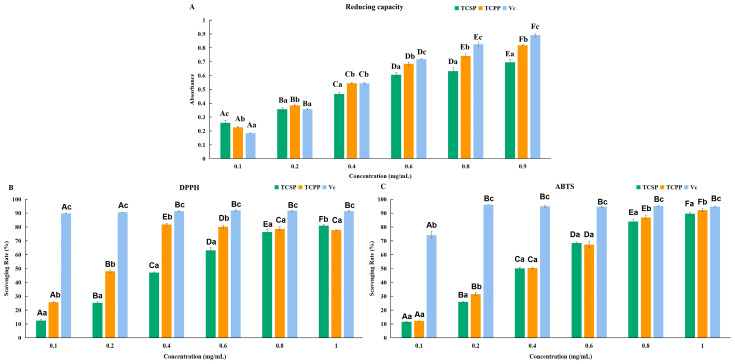
Antioxidant activity of TCPP, TCSP and Vc. Reduction capacity (**A**), Diphenyl picryl hydrazinyl (DPPH) radical scavenging activity (**B**) and 2,2-azino-bis (3-ethylbenzothiazoline–6-sulfonic acid) (ABTS) radical scavenging activity (**C**). Capital letters represent inter-group differences, and lowercase letters represent intra-group differences. TCSP: polysaccharides from stems of *Trollius chinensis* Bunge; TCPP: polysaccharides from petals of *Trollius chinensis* Bunge.

Figure 4A depicts the reducing capacities of TCPP and TCSP compared with a vitamin C (Vc) solution diluted 10 times as a control. The reducing capacities of TCSP and TCPP are dose-dependent, and are notably lower than those of Vc at the corresponding concentrations (Figure 4A). Nevertheless, the reducing capacity of TCPP is slightly stronger than that of TCSP (*p* < 0.05). Some research advocates that the reducing capacity is associated with the presence of reductones that resist oxidation via breaking chain reactions, and there may be a direct correlation between the antioxidant activity and the reducing capacity [28,34]. Although the reducing substances in TCSP and TCPP are less abundant than in those in Vc, the reducing capacities of TCSP and TCPP are stronger compared with those of the polysaccharides of *pumpkin* and *red seaweed*, and are close to that of the extract from *Horsetail aerial* parts [35,36,37].

#### 2.5.2. Diphenyl Picryl Hydrazinyl Radical Scavenging Activity

When the tested dosages were between 0.1 and 1.0 mg/mL, the scavenging activities of all the tested samples were dose-dependent (Figure 4B). Vc shows the best dphenyl picryl hydrazinyl (DPPH) radical scavenging activity. The scavenging rate of TCPP is continuously enhanced until the concentration of 0.4 mg/mL (81.89 ± 0.69%) is reached, and then decreases slightly. In comparison, the DPPH radical scavenging rate of TCSP is steadily increased. Below the concentration of 0.8 mg/mL, the scavenging rate of TCPP is stronger than that of TCSP. When the sample concentration is up to 1 mg/mL, the scavenging rate of TCSP (81.04 ± 1.03%) is stronger than that of TCPP. This result also demonstrates that TCSP have the DPPH radical scavenging effect at the concentration of 1 mg/mL, which is similar to that of TCPP. The half maximal inhibitory concentration (IC_50_) of both TCPP and TCSP is significantly lower than that of Vc (*p* < 0.05) (Table 3). Although the scavenging rate of TCSP is lower than that of TCPP (*p* < 0.05), it still exceeds 80% at 1.0 mg/mL. Compared to other polysaccharides, the DPPH radical scavenging efficiency of TCSP is stronger than that of pumpkin polysaccharides (43.7% at 1.0 mg/mL), and polysaccharides from *Platycodon grandiflorus* (about 18% at 1.0 mg/mL) [38,39]. Meanwhile, the IC_50_ of TCSP (0.5320 ± 0.0497 mg/mL) proves its strong DPPH radical scavenging activity, compared with those of *Osmanthus fragrans* (0.812 mg/mL), *Yerba mate* polysaccharides (1.25 mg/mL), *Stropharia rugosoannulata* polysaccharides (3.11 mg/mL), and potato peel polysaccharide (11.578 mg/mL) [40,41,42,43]. However, the IC_50_ of TCSP is significantly weaker than that of polysaccharides from *Rosa rugosa* petals (about 0.169 mg/mL) [31], or polysaccharides extracted from *Pistachio* external hull (0.08 mg/mL) [44]. The results indicate that TCSP is a potential radical scavenger by exhibiting higher DPPH radical scavenging activity.

#### 2.5.3. 2,2-Azino-bis (3-Ethylbenzothiazoline–6-sulfonic Acid) Scavenging Activity

Figure 4C displays the 2,2-azino-bis (3-ethylbenzothiazoline–6-sulfonic acid) (ABTS) radical scavenging activity of TCPP and TCSP compared with that of Vc as a control. When the tested dosages were between 0.1 and 1.0 mg/mL, the scavenging activities of all tested samples were dose-dependent. Vc also showed the best ABTS radical scavenging effect, and the scavenging rate was maximized to 96.10% ± 0.06% when the concentration was 0.2 mg/mL (Figure 4C). The ABTS radical scavenging rates of TCPP and TCSP similarly increased (*p* > 0.05) until the concentration of 1 mg/mL was reached, and are 92.25 ± 1.17% and 89.59 ± 1.02%, respectively. The IC_50_ values of TCPP and TCSP are remarkably lower than that of Vc (*p* < 0.05, Table 3). Compared to other polysaccharides, the IC_50_ of TCSP on ABTS radicals is stronger than that of potato peel polysaccharides (2 mg/mL), or polysaccharides extracted from *Auricularia auricula* [43,45]. However, the IC_50_ of TCSP on ABTS radicals is weaker than that of polysaccharides extracted from *Pistachio* external hull (about 0.05 mg/mL), or polysaccharides from *Carex meyeriana Kunth* (about 0.03 mg/mL) [44,46]. Moreover, TCSP have comparable ABTS radical scavenging activity to polysaccharides from corn silk [47]. Clearly, the results proved that TCSP are a potential ABTS radical-scavenger.

The above results fully demonstrate that the antioxidant capacity of TCSP is slightly weaker than that of TCPP. Surprisingly, TCSP show similar antioxidant activity to the polysaccharides from corn silk, which is an important and traditional Chinese herbal medicine [47]. The monosaccharide composition of TCSP is generally similar to that of TCPP, except for GlcA and Xyl. Moreover, the Fuc and GalA in TCSP may play vital roles in antioxidation capacities considering the presence of hydroxyl groups and other groups with strong reducibility [26]. The radical scavenging activity of polysaccharides is positively correlated to the hydrogen-donating ability of hydroxyl groups [48,49,50]. 

### 2.6. Comprehensive Comparison of TCSP and TCPP

**Figure 5:**
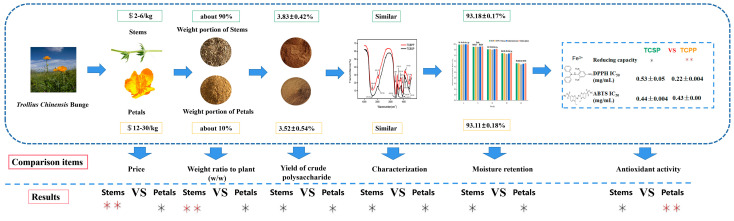
Polysaccharide comparisons of TCSP and TCPP. *: the comparison items are similar, **: the comparison items are better. TCSP: polysaccharides from stems of *Trollius chinensis* Bunge; TCPP: polysaccharides from petals of *Trollius chinensis* Bunge.

The comparison of TCSP and TCPP in terms of the aspects of price, weight ratio to plant, yields of crude polysaccharides, characterization, moisture retention and antioxidant activity is shown in Figure 5. The price of TCS was about 2–6 dollars/kg, which is far below the price of TCP (about 12–30 dollars/kg), the weight portion of TCS accounts for 90% of the total plant weight of TC, which is much higher than the weight portion of TCP to the total plant weight of TC. The yields of crude polysaccharides from TCSP and TCPP were similar, and there was no significant difference. TCS has a low-cost advantage in industrial development and applications. From the results of the analysis of monosaccharide composition and FT-IR, the structures of the two types of polysaccharides are highly similar in terms of the types of monosaccharides in the composition and the types of functional groups. In terms of moisture retention, both TCSP and TCPP show strong moisturizing ability, which is even better than that of chitosan, which is commonly as a humectant in the cosmetics industry. Although the antioxidant activities of TCSP are weaker than those of TCPP, they have stronger antioxidant activities compared to other plant-derived polysaccharides, including pumpkin, red seaweed, potato peel, *Platycodon grandifloras* [39], *Yerba mate* [41], *Stropharia rugosoannulata* [42], and *Auricularia auricula* [45]. Polysaccharides from discarded stems of *Trollius chinensis* Bunge elicit promising potential in the cosmetics or food industries because of their low cost, strong moisture retention and antioxidant activity. Previous research fully confirms that the mixture of crude polysaccharides and crude flavones of a certain proportion has a good synergistic anti-oxidant and moisturizing effect [51]. This result further confirms that TCSP have great application potentials in cosmetics products.

## 3. Materials and Methods

### 3.1. Materials 

The TCS (about 30–80 cm) and TCP were collected from the market in Yakeshi city (47°39′–50°52′ N, 120°28′–122°29′ E), Inner Mongolia Autonomous Region, Northeast China, in 2019. The petals and the stems were dried and ground through a 40 mesh sieve.

### 3.2. Extraction of Crude Polysaccharides from Stems and Petals of TC 

The crude polysaccharides from TCS and TCP were extracted using a water-extraction method. According to a previous study by our team, the best extraction optimum conditions were as follows: a material–liquid ratio of 1:60 (g/mL), a temperature of 80 °C, and duration of 3 h. The extraction was stirred repeatedly 3 times with an SZCL-3B magnetic stirrer (Zhengzhou Biochemical Instrument Co., Ltd., Zhengzhou, China). Then, the solutions from the 3 repetitions were combined and filtered. The filtrate was concentrated to 1/5 of the original volume on a rotation evaporator (DK-98, Tianjin Tiantai Instrument Co., Ltd., Tianjin, China) under 65 ℃ and cooled to room temperature. Then, 95% ethanol (*v*/*v*) was added to the polysaccharide solution until the content declined to 80% (*v*/*v*), and the solution was kept at 4 °C for about 12 h to precipitate the polysaccharides. Then, the precipitate was washed firstly with ethanol at least 3 times, and centrifuged (4000× *g*) for 10 min using a centrifuge (TGL-16M, XiangYi Centrifuge Instrument Co., Ltd., Changsha, China). After removing the proteins using the Sevage method for at least 3 times, the water phase was dialyzed against distilled water about for 3 days to remove salts [52]. Then, the crude polysaccharides from TCS and TCP were extracted. The yield (*w*/*w*) of crude polysaccharide was calculated by Equation (1):

(1) $$Yield(\%)=\frac{M}{W}\times 100\%$$

where *M* is the weight of crude polysaccharides; *W* (g) is the weight of the TC powder. 

### 3.3. Chemical Composition

The total carbohydrate content of the crude polysaccharide powder was measured with the phenol-sulfuric acid method [53]. The uronic acid content was confirmed with a meta-hydroxydiphenyl assay [54]. The total flavonoid content was measured spectrophotometrically using a modified colorimetric method by Lu et al. [3]. The protein content was measured by the Bradford assay [27].

### 3.4. Purification of Crude Polysaccharides

The crude polysaccharides were purified by ion exchange chromatography using DEAE-cellulose (2.6 × 30 cm) [28]. 

### 3.5. Molecular Weights and Analysis of Monosaccharide’s Compositions 

The molecular weight distribution of purified polysaccharides was measured via high-performance size exclusion chromatography (HPSEC-RID). Standard dextran samples (National Institute for Food and Drug Control, Beijing, China) with different molecular weights (36,800, 64,650, 135,350, 300,600, and 2,000,000 Da) were determined under the same conditions to establish a calibration curve [46].

The monosaccharide compositions of purified TCSP and TCPP were analyzed with a high-performance liquid phase-evaporation light scattering detector (HPLCELSD) [46,53]. The monosaccharide samples of Man, Rib, Rha, GlcA, GalA, Glu, Gal, Xyl, Ara and Fuc were mixed together as the test standard sample. [1,10,55].

### 3.6. FT-IR Ananlysis of TCSP and TCPP

The purified TCSP and TCPP were analyzed by an FT-IR spectrophotometer (Gangdong SCI. & TECH. Development Co. Ltd., Tianjin, China) [32,56]. 

### 3.7. Measurement of Moisture Absorption and Retention Abilities

The crude polysaccharides (TCSP and TCPP), and other common humectants in cosmetics (e.g., chitosan, sodium alginate and sodium hyaluronate) were selected. The samples were all oven-dried for 4 h at 100 °C and then placed in a sealed humidity chamber with a saturated Na_2_CO_3_ solution (43% RH) and a saturated (NH_4_)_2_SO_4_ solution (81% RH) at 25 °C for the indicated time [57,58]. The moisture absorption ability (Ra) was measured as follows:

(2) $$Ra\left(\%\right)=\frac{W_{1}-W_{0}}{W_{0}}\times 100\%$$

where *W*_0_ and *W*_1_ are the sample weights before and after being put into the chamber, respectively. 

For the evaluation of moisture retention, 20 mL of distilled water were added to the samples, which were then put into a silica gel (43% RH) desiccator at 25 °C for the indicated time [59]. The moisture retention rate (Rh) was measured as follows:

(3) $$Rh\left(\%\right)=\frac{H_{1}}{H_{0}}\times 100\%$$

where *H*_0_ and *H*_1_ are the water weights in the sample before and after being put in the desiccator at 25 °C, respectively. 

### 3.8. Antioxidant Activity In Vitro

#### 3.8.1. Determination of Reducing Capacity

According to the research of Lu et al. [3], the method of determining the reducing capacity was not elaborated here.

#### 3.8.2. DPPH Radical Scavenging Activity

Based on the research of Chen et al. [30], the method of DPPH was not described in detail here. The scavenging rate was calculated as follows:

(4) $$\mathrm{Scavenging}\;\mathrm{Rate}(\%)=\frac{A_{0}-A_{1}}{A_{0}}\times 100\%$$

where *A*_0_ and *A*_1_ are the absorbance values of the blank sample and the test sample, respectively.

### 3.9. ABTS Radical Scavenging Activity

The method of ABTS detection can be found in the research of Hu et al. [60]. The scavenging rate was calculated according to Equation (4). Vitamin C (Vc) was used as a control.

### 3.10. Statistical Analysis

All the data were represented as mean ± standard deviation (SD) based on 3 parallel tests. Statistical differences were analyzed via a one-way analysis of variance (ANOVA) followed by Duncan’s multiple range test to determine significant differences or a *t*-test on SPSS 17.0. *p* < 0.05 was considered to be statistically significant.

## 4. Conclusions

TCSP have lower M_n_ and M_w_, and wider molecular weight distribution (PDI = 3.55) than those of TCPP. TCSP have a generally similar monosaccharide composition to that of TCPP, but GlcA and Xyl only exist in TCSP. The FT-IR spectra prove there may be intermolecular hydrogen bonding and carboxyl groups in the polysaccharide structure. There is no difference in the moisture retention ability between TCSP and TCPP, which are both highly homologous with traditional humectants, including sodium hyaluronate, sodium alginate and chitosan. The anti-oxidation assays in vitro demonstrated that although the antioxidant activities of TCSP are weaker than those of TCPP, TCSP has stronger antioxidant activities compared to other plant-derived polysaccharides, including pumpkin, red seaweed, potato peel, Platycodon grandiflorus, Stropharia rugosoannulata, Yerba mate, and Auricularia auricula. These results prove that the stems of TC can be a promising source of unconventional polysaccharides, which possess moisture retention and antioxidation capacities for the cosmetics industry. This discovery will greatly improve the comprehensive application value of TC and avoid waste.

## Figures and Tables

**Table 1:** Yield and main components of polysaccharides from stems and petals of *Trollius chinensis* Bunge (mean ± standard deviation).

Sample	Yield (%)	Total Sugar Content (%)	Uronic Acid (%)	Flavonoids (%)	Protein (%)
TCSP	3.83 ± 0.42 ^a^	56.99 ± 8.12 ^a^	7.56 ± 0.45 ^a^	8.06 ± 1.82 ^a^	0.92 ± 0.24 ^a^
TCPP	3.52 ± 0.54 ^a^	46.09 ± 1.68 ^a^	4.76 ± 0.81 ^b^	9.52 ± 3.42 ^a^	0.85 ± 0.21 ^a^

TCSP: polysaccharides from stems of *Trollius chinensis* Bunge; TCPP: polysaccharides from petals of *Trollius chinensis* Bunge. Different superscript letters in each column indicate significant t-test differences between TCSP and TCPP *(p* < 0.05).

**Table 2:** Molecular weight and monosaccharide composition of TCSP and TCPP.

Sample	M_n_ 1 × 10^5^ Da	M_w_ 1 × 10^5^ Da	PDI (M_w_/M_n_)	Monosaccharide’s Composition (% mol)								
				Man	Rha	GlcA	GalA	Glu	Gal	Xyl	Ara	Fuc
TCSP	1.71	6.07	3.55	10.10 ± 0.23 ^b^	3.11 ± 0.04 ^a^	3.09 ± 0.07	30.29 ± 3.72 ^b^	13.36 ± 1.2 ^a^	11.16 ± 0.86 ^a^	6.33 ± 0.59	14.78 ± 2.33 ^a^	7.79 ± 0.73 ^a^
TCPP	7.33	9.72	1.33	7.81 ± 0.16 ^a^	5.04 ± 0.12 ^b^	ND	5.13 ± 2.38 ^a^	28.15 ± 2.36 ^b^	14.98 ± 1.03 ^b^	ND	16.60 ± 2.09 ^b^	13.29 ± 1.15 ^b^

Molar ratio of monosaccharides based on the percentage of their peak area. Different superscript letters in each column indicate significant differences by one-way analysis of variance (*p* < 0.05). TCSP: polysaccharides from stems of *Trollius chinensis* Bunge; TCPP: polysaccharides from petals of *Trollius chinensis* Bunge. M_n_: number-average molecular weight; M_w_: weight-average molar masses; Man: mannose; Rib: ribinose; Rha: rhamnose; GlcA: glucuronic acid; GalA: galacturonic acid; Glu: glucose; Gal: galactose; Xyl: xylose; Ara: arabinose; Fuc: fucose. ND: not detected.

**Table 3:** IC_50_ of TCSP and TCPP (mean ± standard deviation).

Sample	DPPH-IC_50_ (mg/mL)	ABTS-IC_50_ (mg/mL)	Sample
TCSP	0.5320 ± 0.0496 ^a^	0.4390 ± 0.0040 ^a^	TCSP
TCPP	0.2193 ± 0.0061 ^b^	0.4273 ± 0.0035 ^a^	TCPP
Vc	0.0034 ± 0.0002 ^c^	0.0026 ± 0.0002 ^b^	Vc

IC_50_: half maximal inhibitory concentration; TCSP: polysaccharides from stems of *Trollius chinensis* Bunge; TCPP: polysaccharides from petals of *Trollius chinensis* Bunge. Different superscript letters in each column indicate significant differences among samples by one-way analysis of variance (*p* < 0.05).

## Data Availability

Data are contained within the article.

## Supplementary Materials

The following supporting information can be downloaded at: https://www.mdpi.com/article/10.3390/molecules28073114/s1.
