# Supplementary material for: Polysaccharides from Discarded Stems of Trollius chinensis Bunge Elicit Promising Potential in Cosmetic Industry: Characterization, Moisture Retention and Antioxidant Activity
Source: Molecules. 2023 Mar 30;28(7):3114. doi: 10.3390/molecules28073114 (PMC10095818; doi:10.3390/molecules28073114)
Supplement: Supplementary file 1 [file molecules-28-03114-s001.zip › molecules-2229878-supplementary.pdf]

Supplementary File

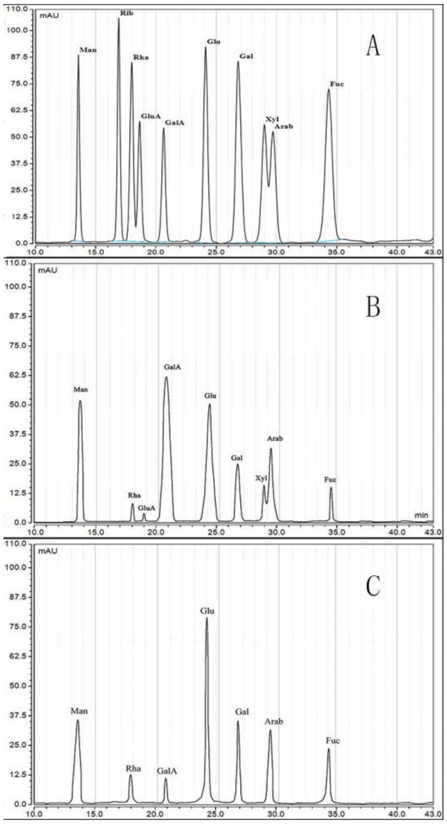

Figure S1. Monosaccharide analysis of standard sample (**A**), TCSPs (**B**) and TCPPs (**C**) with high performance liquid chromatography.
